# An operational guide to translational clinical machine learning in academic medical centers

**DOI:** 10.1038/s41746-024-01094-9

**Published:** 2024-05-17

**Authors:** Mukund Poddar, Jayson S. Marwaha, William Yuan, Santiago Romero-Brufau, Gabriel A. Brat

**Affiliations:** 1 Department of Biostatistics, Harvard T. H. Chan School of Public Health, Boston, MA USA; 2 Department of Surgery, Beth Israel Deaconess Medical Center, Boston, MA USA; 3 Department of Biomedical Informatics, Harvard Medical School, Boston, MA USA; 4 Department of Otolaryngology Head & Neck Surgery, Mayo Clinic, Rochester, MN USA

**Keywords:** Translational research, Research data

## Abstract

Few published data science tools are ever translated from academia to real-world clinical settings for which they were intended. One dimension of this problem is the software engineering task of turning published academic projects into tools that are usable at the bedside. Given the complexity of the data ecosystem in large health systems, this task often represents a significant barrier to the real-world deployment of data science tools for prospective piloting and evaluation. Many information technology companies have created Machine Learning Operations (MLOps) teams to help with such tasks at scale, but the low penetration of home-grown data science tools in regular clinical practice precludes the formation of such teams in healthcare organizations. Based on experiences deploying data science tools at two large academic medical centers (Beth Israel Deaconess Medical Center, Boston, MA; Mayo Clinic, Rochester, MN), we propose a strategy to facilitate this transition from academic product to operational tool, defining the responsibilities of the principal investigator, data scientist, machine learning engineer, health system IT administrator, and clinician end-user throughout the process. We first enumerate the technical resources and stakeholders needed to prepare for model deployment. We then propose an approach to planning how the final product will work from data extraction and analysis to visualization of model outputs. Finally, we describe how the team should execute on this plan. We hope to guide health systems aiming to deploy minimum viable data science tools and realize their value in clinical practice.

## Introduction

**Fig. 1:**
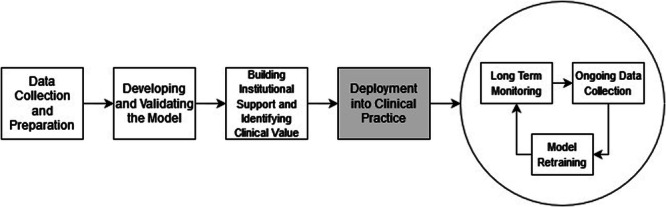
The pipeline for making usable ML solutions; the focus of this piece is highlighted in gray.

In recent years, researchers have contributed innumerable data science tools - clinical risk prediction models, medical image classification algorithms, and more - to the medical literature. Only a small fraction of these tools, an estimated 10% or less, have been implemented in real-world clinical settings where they can improve patient care^1^. Several explanations have been proposed to explain this gap including limited external validity, actionability, and reproducibility of published data science tools^2–4^. One infrequently discussed reason is the difficulty of putting these models, often developed in research laboratories, into a production environment where they can consistently run in real-time (Fig. 1). The act of transforming academic projects into real-world instruments that end users such as physicians and nurses can access and leverage at the bedside is a non-trivial software engineering task in large health systems.

Other industries have addressed this problem by building teams, resources, and protocols for translating promising *in-silico* academic products into real-world practice. Many software companies have “R2O” (research to operations) or “MLOps” (machine learning operations) teams devoted to this task. Deployment in healthcare settings presents a unique challenge because organizational resources for this task are often constrained and the clinical data that powers these tools is often 1) fragmented across multiple IT systems, 2) not easily accessible from the organization’s data warehouse, 3) not captured in real-time or missing, or 4) lacking structure. While online tools like MDCalc and similar standalone smartphone applications have been used to make risk calculators accessible to providers at the point-of-care, applications lacking deep electronic health record (EHR) integration are not suitable for complex ML models that require a significant number of inputs and computing power to generate predictions. Some EHR providers provide a framework to allow easy integration for ML models, but healthcare institutions may not use them or find them unsuitable and require more bespoke solutions.

This piece, assembled by a team of clinicians, informaticians, data scientists, and software engineers that deployed academic projects into production at multiple large health systems, aims to provide some guidance to the deployment team on how to best make that translation. We believe that the full team involved in the deployment would benefit from having an understanding of the different roles and tasks that are often required, but it is probably the principal investigator or team champion that would benefit the most from the high-level view that we present in this manuscript. Throughout the paper we reference an example of translating a prediction model into clinical practice from the authors. This model generated predicted risk of surgery within the next 6 months for chronic colon inflammation patients seen in a gastroenterologist’s office.

Of note, this document is focused on the technical aspects of translating research algorithms for clinical practice. There are numerous additional steps that are not covered herein. Important elements include identifying and proving clinical utility of a model, its development, and regulatory requirements that often strongly impact an intervention’s scope, pace, and form. In parallel to the development process described, the deployment and clinical team should address the needed legal and policy requirements of their institution.

Additionally, deployment of data science solutions into clinical practice is often an iterative process that requires multiple feedback loops between the development team and the end-user representatives at different levels, including retraining after deployment due to gradual changes to standard of care or abrupt shocks like COVID-19^5^. For simplicity, we present a linear process, but it is strongly advised to incorporate the flexibility for iteration at different points. The iterative nature of the process also highlights one of the reasons why it’s important to be thoughtful when building deployment infrastructure.

## Prerequisites for model deployment

The purpose of this guide is to help groups within health systems implement tools that they already have the interest and resources to use and support. Deployment should not be considered until the tool has, at least, nominal institutional support. Details on how to meet these prerequisites can be found elsewhere but are briefly summarized below^2^.

### Clinical value proposition

Before deployment is considered, the value of a model must be explicitly articulated and aligned with the priorities of the organization. Impacting elements of the Institute for Healthcare Improvement Quadruple Aim is a good benchmark for identifying clinical value^6^. A pathway to achieving this value should be clearly identified including what clinical decision the model will impact (e.g. recommending curative surgery), what outputs need to be delivered to the user (e.g. probability of outcome and visualizations of prognosis for similar patients), who is the target of output (e.g. gastroenterologist), when it will be delivered (e.g. during an outpatient encounter), how users can take action on the model’s output (e.g. recommend a consult to the colorectal surgeon), and the target population (e.g. patients with moderate-to-severe ulcerative colitis). Depending on the training dataset’s design, the model might need to be fine-tuned to local clinical practice and population.

### Stakeholders

The translation process is multidisciplinary by nature, and different stakeholders required to implement the model into production should have opportunities to discuss their unique perspectives together (Table 1). Multiple roles might be taken on by the same person based on skills and technical complexity in the context of the project, but a team should include the following roles:

- Principal Investigator/Project Champion who understands the clinical and institutional context
- Data Scientist who knows how the model was built
- Machine Learning Engineer (MLE) who will put the model into production
- Clinicians/Users who will best know what is required for the model outputs to be useful
- Data engineers/Information Technology team who understand the data sources and EHR vagaries

**Table 1:** Index card for major stakeholders and their tasks

Role	Main responsibility	Tasks
Principal Investigator	Maintain an overview of the project and coordinate tasks.	Identify and convene relevant stakeholders for the project Ensure smooth information flow among stakeholders across different project phases and modules Review that complete set of information is transferred during stage transitions Vet the infrastructure constraints Approve interface mock-ups Weekly oversight of project timeline and budget
Machine-learning engineer	Program a tool that pulls data, generates a prediction and delivers it to the end user according to the needs	They are the primary drivers for all tasks in the paper
Data scientist	Ensure the MLE is able to faithfully transcribe the model into a working platform and modify the model as required	Document the modeling process: data sources, pre-processing steps, model inference, outputs and interpretation Make changes to the model based on evolving needs
Clinician or user representative	Ensure the model output is valuable to the end user	Confirm availability of model inputs at time of prediction generation Ensure interpretability of model outputs and their relevance given clinical needs Provide feedback on application interface
Information Technology	Serve as technical expert on data sources, infrastructure hosting and best practices	Help connect to and pull data from the data warehouse Vet hardware constraints for the platform Help host the platform

The various team members require constant communication given the interwoven roles and expertise needed to deploy a complex tool. The MLE typically has limited insight into the decisions made earlier or the model development, most often by a clinical developer and data scientist. A data scientist may not know technical requirements and best practices necessary for model deployment. A data engineer primarily cares about data security and limiting stress to the data infrastructure and may not prioritize the project. The principal investigator needs a global view of the process to know what challenges are involved and how to budget time. Users have domain knowledge, but may not understand or care for technical details. The model needs to be interpretable by them given their requirements and context.

### Timely data availability

Necessary data inputs at the health system should be available on or before the time that the prediction is needed. For example, if the point-of-care model uses an encounter’s prescription medication as an input, there needs to be a process to capture such data during the encounter itself. In the case of our ulcerative colitis model, we had users input this data via a form, as prescription data was not available in real-time from other sources. Of course, data sources that directly integrate into the clinical workflow are preferable, but may often not be available during early stages or stepwise clinical integration.

### Operational home

Although it may not always be possible in advance, it is important to plan for the maintenance of the implemented model and identify an operational home where a team takes responsibility for uptime, at least for the current anticipated project horizon (e.g. during the initial pilot). Stakeholders should be available as needed for the project’s initial duration to tackle data science problems (such as performance degradation and dataset drift - see “Post-Deployment Considerations” section for more details on this)^5^ and changes to clinical workflows.

## Planning for deployment

Prior to building a deployable tool, it is essential for the MLE to have a complete understanding of the requirements and specifications of the desired end product. This is only possible once all prerequisites in the prior section have been met, as each stakeholder provides distinct and complementary information on the vision for the final product.

We propose that the MLE approach the task of gathering requirements or specifications in a *inverted* extract, transform, load (ETL) fashion (Table 2). Many digital health tools follow the standard ETL framework, where data is extracted from the source (e.g. the EHR), transformed in some clinically meaningful way, and loaded onto a dashboard for clinicians to view. In contrast, working backwards may be helpful in the planning and designing phase. First, clinician end-users should be engaged to understand the tool’s purpose and what information needs to be loaded into the tool to fulfill its purpose. Then, the data scientists and PI should be engaged to understand what data inputs and transformations are needed to generate this desired information. Finally, the PI should engage the health system’s data and IT engineers to map the necessary inputs to data elements collected by the health system and identify where they reside in the organization’s data repository. This is an iterative process that requires multiple discussions with each stakeholder.

**Table 2:** Specific questions to address in inverted extract, transform, load (ETL) order before the tool-building process is begun

Inverted-ETL Phase	Consideration	Example 1:	Example 2
Load	What clinical question will the tool answer?	Clinicians sought a tool that would help them decide whether an IBD patient would benefit from an elective colectomy.	Clinical teams wanted a tool to help identify deteriorating patients in real-time and facilitate coordination (e.g.., nursing, physicians, APPs).
	In what clinical scenario will it be used?	The tool was designed for use when evaluating a patient in the office.	The tool will be active for inpatient general care floors.
	What information is useful to the end-user, and how should it be presented?	Clinicians specifically wanted to know: 1) the predicted likelihood that a patient would need a colectomy within the next 6 months 2) their illness severity trajectory over the last few office visits 3) their projected risk of surgery over time 4) what typically happens to patients with similar illness severity. We iterated over several wireframe diagrams and mockups with clinician, PI, and data scientist input.	The final design that was implemented included alerts sent in real-time to nursing and provider pagers with the following information: 1) Which patients were at risk of deterioration (clinic number, patient room) 2) who had been alerted. Care team members were expected to communicate with each other and evaluate the patient. Follow-up alerts were sent if the patient’s risk continued to be elevated.
	Who will access the information?	The tool should be accessible to gastroenterologists and colorectal surgeons within our health system who evaluate IBD patients; it should not be accessible to anyone beyond our institution. Therefore, we chose to host our tool on a hospital server within its firewall.	The information was delivered to the nurse and providers taking care of the patient (primary RN, charge RN, service pager, and eventually attending pager).
Transform	What data outputs does the model provide?	The model provides the predicted likelihood that a patient will need a colectomy within the next 6 months.	The model only provided binary information: an alert if the patient was considered at risk.
	What data inputs are needed to produce the desired output?	The model takes a patient’s current and past medication history, demographics, comorbidities, procedures, and recent symptoms as inputs.	The model used clinical data as predictors (vital signs, labs, medications, nursing evaluations, etc.), as well as other information to filter alerts (e.g., “comfort cares only” status, patient location).
	What is the required time between the data being generated and the outputs being presented to the user?	For our model, most data inputs were readily available in the hospital’s data warehouse except for prescription medication changes made at the point of care. Therefore, this data was captured by directly asking the clinician to enter what they prescribed.	This was a time-sensitive use case, the maximum delay we allowed was in the order of 15 minutes, so the system would run every 15 minutes.
Extract	What data elements collected by the hospital correspond to the inputs needed?	Encounter details, medication history, demographics, procedures, comorbidities, and symptoms are encoded in various clinical terminologies (e.g., ICD-10, RxNorm, CPT).	Some examples: there were around 10 different variables for blood pressure (depending on measurement type, patient position, etc.) Patient location was determined using 2 different system (the administrative Admission-Discharge-Transfer system as well as the location associated with automatic vital signs measurements).
	Where are these data elements found in the hospital’s data warehouse?	We had several meetings with IT and database administrators at our health system to identify the tables that contained our desired data elements.	The data elements needed to run the final production system (both for prediction and for alert filtering) were stored in several different systems and had to be coordinated.

Two examples are provided from the authors’ experience deploying a surgical prediction model for ulcerative colitis patients (Example 1) and a prediction model for inpatient clinical deterioration among general medical/surgical patients (Example 2)^15^.

Another important task at this stage is for the PI to decide whether this tool’s deployment process should lay the groundwork for deployment of future data science tools as well by establishing a data pipeline, or instead serve as a bespoke process with the goal of simply bringing this one tool to production. Platforms meant to support multiple future data science tools should be built with modules that are reusable^7^, have the ability to take in more data as parameters instead of hardcoding, and allow flexibility for extensions. Building platforms requires more time, coding, careful design and experience, so for teams that are attempting their first model deployment, our recommendation is to start by limiting the scope of deployment to the initial project. Over subsequent iterations, the tool can be evolved into a platform.

The end product of this preparation phase should be an architecture diagram that enumerates the technologies, frameworks, tools, and connections powering the tool and its underlying data flow. This includes any cloud technologies and data stores used, wireframe diagrams, and mock screenshots of the end application.

## Building a deployable tool

### General Infrastructure considerations

With an inverted-ETL deployment plan in place, the team is now ready to deploy the tool. This section provides step-specific guidance to the ML engineer on this process, from pulling data, to processing data into the model, to presenting model outputs. Throughout the process of deployment, the following issues below should remain in mind and be addressed at each step.

#### Infrastructure selection

An option of cloud infrastructure, hosted infrastructure, native infrastructure, or some combination thereof can be considered to run the complete application. This decision is strongly influenced by overarching organizational requirements and existing infrastructure. For example, at one of the authors’ institutions, on-premises cloud infrastructure is strongly recommended, and there is little to no support for other types of infrastructure. However, if multiple options are viable, choosing cloud infrastructure would be most preferable as it would help manage usage volume uncertainties during the pilot and provide turnkey support for security and best practices^8^.

#### Information security

Information security and the corresponding committee approvals that the institution may require should be a longitudinal consideration instead of a costly afterthought. In the past, failure to get data security clearance has caused us to significantly delay go-live. At each stage of the system, only the minimum necessary amount and type of data should be captured and made available. Access authorization might be necessitated depending on the sensitivity of the application, in which case being able to use the existing organizational IT will be most helpful.

#### Best practice adherence

Following standard software development best practices of baking in debuggability, logging, and documentation in the code will lead to a more sustainable and monitored system over time. During the design and build phases, the MLE should make note of critical software units where tests are required to ensure functionality^8^. Intermediate outputs for our batch processing were persisted on disk storage along with summary statistics like counts and means of important features being written to logs. Much thought was put into descriptive error messages for code exceptions for debuggability.

### Generating inputs (pulling and connecting the data)

The goal of this module is to convert a query that was run once to create the training dataset into a reusable pipeline delivering data to the model. The major stakeholders for this module are the data engineer, the data scientist, and the MLE. The data scientist contributes the following information:

1. Data Sources: A list of the databases and tables required.
2. Query to Generate Dataset: The queries used on tables to extract data pertaining to the project. For privacy and security purposes, only the strictly required data should be accessed with the query.
3. Data warehouse contacts/documentation: The tasks will require knowledge about accessing the databases in a structured manner. Any documentation that can help learn about it or contact details for people who can serve as a resource helps keep in check the engineering effort.

This stage requires the data engineer and the MLE to complete the following tasks:

1. Create the Infrastructure to run this periodically/in real-time: The ideal infrastructure should be able to pull data during run time using an Application Programming Interface (API). However, the existing infrastructure might not accommodate APIs, in which case a script should be able to download data at a predetermined frequency (hourly/daily/weekly), and subsequently trigger the pipeline that consumes the information.
2. Identify credentials and software/drivers to use the infrastructure: Different databases will require different drivers and libraries to interface with the programming language of your choice.

### Inputs to outputs (processing the data into the model)

The goal of this module is to make a model work within identified resource and time constraints. The major stakeholders for this module are the MLE, data scientist, and the end users or a product manager. The team needs the following information and artifacts for this module’s development:

1. Preprocessing Code: The code that takes the raw data from the database and makes it into the form required for the model. Also included are checks on the data validity and completeness.
2. Model: The program that feeds the chosen model the inputs and post-processes the outputs as required for further consumption. For a batch processing system, this needs to output a file that can be consumed by downstream systems.
3. Input file examples: An example set of inputs that can be expected for the model. This allows for integration tests from the very start.
4. Output file examples: The expected outputs for the above set of inputs. This is required to ensure results from the integration tests.
5. Tests: Unit tests^9^ for the pre-processing and post-processing modules and validation datasets for the model itself to detect changes in model accuracy. Without tests, any step in this process could result in undetected errors that cascade through the system and become progressively harder to detect. Tests also serve as effective guardrails for the MLE to ensure that each software component they write fits into the intended vision for the data model conceptualized by the data science team. Given the lack of tests during the model development phase, the MLE might also be required to write the tests.

This stage requires the MLE to complete the following tasks with help from the data scientist and health system IT experts:

1. Define Constraints: Various constraints will apply to the model’s running that need to be adhered to for practical reasons. Some of them relate to the hardware available (computing power available, GPU requirements), running time (need to make predictions in less than 3 seconds from user input), scalability (number of parallel requests to be handled) and reproducibility (a set of user inputs should always return the same prediction).
2. Ensure model meets constraints: In an interactive process, the system needs to be developed, benchmarked, and modified to fit in above constraints.
3. Make desired logic changes: As the application is developed and becomes more usable by an end user, stakeholders will request modifications to the underlying model or for different pieces of data to be shown or obscured. Without a robust suite of tests, this is the hardest task for an MLE as they will have low confidence in making changes to code they did not write or completely understand. It is thus also the task determining a model’s long term adoption.

### Outputs to insights (how to present the model outputs)

Over this stage, we aim to make the model usable by end users. This might mean creating just a metric or notification visible on an existing system, or building a dashboard accessible through other systems. Based on the interface desired, a team consisting of a web/mobile app developer, health system IT specialist, machine learning engineer, data scientist, and end users might be required. The team needs to decide the level of EHR interface integration desired and whether mobile devices need special support. The end users need to specify what information (metrics, notifications, graphs, etc) they need through the app to ensure a usable and useful interface is created. While more information being revealed is generally desired, over-exposure to details drives down adoption^10^. While any successful adoption requires seamless integration with existing EHR systems, a pilot program could benefit from a quick but usable de-novo front-end before investing into further EHR integration. The following tasks will help create a clean, modular, and re-usable application:

1. Create mock-ups for the interface: A rough wire-frame diagram helps the end-users and developers align on the vision for the application interface. This should incorporate design guidelines and cues from existing apps in the healthcare system to prevent friction for users (Fig. 2).

**Fig. 2:**
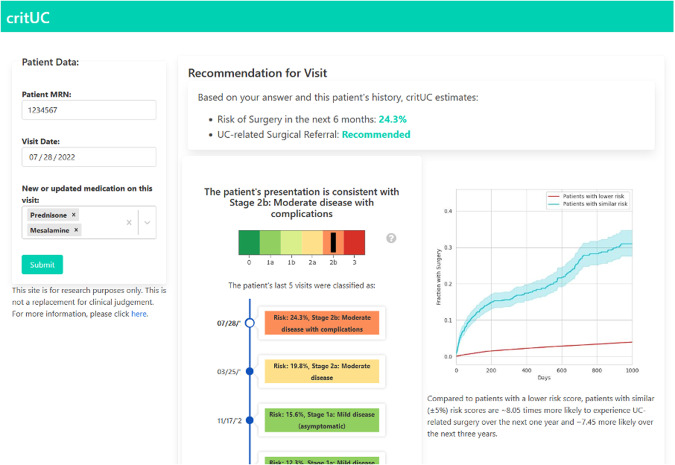
Screenshot of our application showing surgical referral for a patient.
2. Create backend APIs for serving multiple front-ends: The backend is a platform that should allow for different front-ends to be bolted on without requiring any changes. An API-oriented framework for the platform helps with this as well as scalability and testing^11,12^.
3. Make a scalable, portable, reliable app: The application should be able to scale with realistic adoption numbers and be portable enough to be moved with trivial human effort. A reliable application is very important for healthcare settings as clinicians cannot rely on a system that might go down at crucial junctures or fails in unexpected and drastic ways. Standard software development best practices should be adhered to for a truly usable system. These also allow the back-end to be integrated with the EHR front-end easily if the pilot is successful^13^.
4. Front-end to serve the most important use-case: While building this, the team should choose an appropriate tech stack to allow code reusability when being adopted into the EHR in case of a successful pilot program.

### Post-deployment considerations

Once an application is developed and deployed, a pilot program would help validate the application and its clinical value. A pilot that is successful in demonstrating measurable clinical value should then be followed by planning for long-term maintainability to ensure adoption and ongoing use. A detailed explanation of long-term issues to keep in mind such as dataset shift and performance degradation can be found elsewhere^14^, but a handful of the primary concerns worth highlighting here are:

1. Dataset or model drift: Healthcare data, like all real-world data, are subject to fundamental changes that alter their expected distributions, a phenomenon known as dataset shift^5^. When there is a discrepancy between the distribution of the data a model was trained on and the one it is implemented on, performance suffers. One example of this phenomenon was the introduction of new ICD-10 diagnosis codes during the coronavirus pandemic. Prediction models trained on diagnosis code datasets prior to the pandemic saw their performance suffer from 2020 onwards as the distribution of codes significantly changed, as did the fundamental relationships between codes. Dataset shift can also be introduced by changes in technology, policy, behaviors, or demographics^5^. When this is suspected, the data scientist should investigate if the new population is different from the training population and consider re-training the model.
2. Lost data connections: This should be suspected when either the data source breaks down, or the structure of the data input changes (for example, the way medications are recorded changes). The machine learning engineer should be attentive to such changes after deployment.
3. Workflow or clinical practice changes conflict with the system: for example, a new treatment is developed as an alternative to surgery that changes the decisions the model was focused on. Alternatively, if new relevant inputs appear (e.g. a new drug that can reduce the expected need for surgery). The PI or clinical champion should remain on the lookout for such paradigm shifts in care post-deployment and adjust the model accordingly.

## Conclusion

Translating academic data science tools into production in large health systems is a complex task that requires the engagement of multiple stakeholders within the organization. This piece is intended to guide health systems aiming to deploy a minimum viable data science tool for the first time; once an organization has enough tools in production, it may justify investment in building platforms and assembling Machine Learning Operations teams dedicated to this task. The Office of the National Coordinator for Healthcare IT (ONC) Final Rule, which required health systems to make Fast Healthcare Interoperability Resources (FHIR)-based application programming interfaces (APIs) available for transmitting and retrieving clinical data as of December 31, 2022, was an encouraging development that will hopefully make the US healthcare data ecosystem more interoperable and thereby make it easier to deploy such tools. In the meantime, research teams should keep this task in mind from the beginning to maximize the likelihood that their efforts are eventually used to improve patient care.
